# Cocoa by-products: A comprehensive review on potential uses, waste management, and emerging green technologies for cocoa pod husk utilization

**DOI:** 10.1016/j.heliyon.2024.e35537

**Published:** 2024-07-31

**Authors:** Satria Bhirawa Anoraga, Rosnah Shamsudin, Muhammad Hazwan Hamzah, Suzannah Sharif, Arifin Dwi Saputro

**Affiliations:** a Department of Process and Food Engineering, Faculty of Engineering, Universiti Putra Malaysia, Serdang, 43400, Selangor, Malaysia; b Department of Bioresources Technology and Veterinary, Vocational College, Universitas Gadjah Mada, Yogyakarta, 55281, Indonesia; c Institute of Plantations Studies, Universiti Putra Malaysia, Serdang, 43400, Selangor, Malaysia; d SMART Farming Technology Research Centre, Department of Biological and Agricultural Engineering, Faculty of Engineering, Universiti Putra Malaysia, Serdang, 43400, Selangor, Malaysia; e Cocoa Innovation and Technology Centre, Malaysian Cocoa Board, Lot 12621, Nilai Industrial Area, Nilai, 71800, Negeri Sembilan, Malaysia; f Department of Agricultural and Biosystems Engineering, Faculty of Agricultural Technology, Universitas Gadjah Mada, Yogyakarta, 55281, Indonesia

**Keywords:** By-products, Cocoa pod husk, Potential uses, Waste management, Green technologies

## Abstract

Cocoa is considered to be one of the most significant agricultural commodities globally, alongside Palm Oil and Rubber. Cocoa is the primary ingredient in the manufacturing of chocolate, a globally popular food product. Approximately 30 % of cocoa, specifically cocoa nibs, are used as the primary constituent in chocolate production., while the other portion is either discarded in landfills as compost or repurposed as animal feed. Cocoa by-products consist of cocoa pod husk (CPH), cocoa shell, and pulp, of which about 70 % of the fruit is composed of CPH. CPH is a renewable resource rich in dietary fiber, lignin, and bioactive antioxidants like polyphenols that are being underutilized. CPH has the potential to be used as a source of pectin, dietary fibre, antibacterial properties, encapsulation material, xylitol as a sugar substitute, a fragrance compound, and in skin care applications. Several methods can be used to manage CPH waste using green technology and then transformed into valuable commodities, including pectin sources. Innovations in extraction procedures for the production of functional compounds can be utilized to increase yields and enhance existing uses. This review focuses on the physicochemical of CPH, its potential use, waste management, and green technology of cocoa by-products, particularly CPH pectin, in order to provide information for its development.

## Highlights

• Cocoa pod husk can be potentially used as a pectin source.
• Freshly cut cocoa pod husk had organic matter, which consisted of fiber.
• Integrated system is needed to transform waste into valuable compounds.
• Green techniques promise higher yields of pectin and shorter time.
• Waste utilization aligns with the SDGs and support zero hunger.

## **1** Introduction

Chocolate is one of the most popular confectionary products in the world. The main ingredient for chocolate is cocoa. Cacao is massively grown in West Africa, south America, and south Asia. Global cocoa production continues to increase. In 2020, global cocoa production reached 4735 thousand tons, with the highest producer being Africa, followed by America and Asia-Oceania [1,2]. The mean yearly cocoa production in Malaysia is from 0.8 to 1.0 metric tonnes per hectare, whereas in Brazil and Indonesia, the corresponding figures are 0.5–0.6 metric tonnes per hectare [3].

The basic problems associated with this production process are the amount of organic waste produced continuously. There is a wide range of biomass waste that includes agricultural wastes, fruit processing wastes, and wastes from the processing of other food in manufacturing sector, including chocolate production and cocoa processing which encompasses approximately 100 billion metric tons of biomass waste that are generated annually in the world [4]. Inadequate management of cocoa waste can cause significant environmental damage, financial losses, and health problems [5].

Cocoa pod husk (CPH), along with other undesirable by-products such as cocoa shells and pulp, are significant by-products and account for approximately 70–80 % of the whole cocoa fruit weight [6]. The cocoa bean is the only component of the cocoa plant that is utilized in the production of chocolate [7]. Commonly, cocoa by-products are often simply discarded or utilized as fertilizer by burying them in the soil [8]. However, cocoa by-products hold immense potential as a valuable source of raw materials for extracting essential substances [9]. Moreover, this type of residual biomass is readily accessible and offers cost-effective acquisition, rendering it highly appealing to the industrial sector [10]. Numerous effective alternative approaches exist for managing residues from cocoa plants, with biotechnology standing out as a method aimed at minimizing the accumulation of substantial residue in the soil [11].

Effective waste management is critical in attempts to prevent food waste and develop circular economy concepts [12]. Waste management approaches generally include the reuse and valorization of by-products through innovative processes, their utilization as functional ingredients rich in bioactive compounds, and the extraction of valuable active ingredients [13,14]. Plant-derived biomass waste is a sustainable natural resource that contains phytochemicals [15]. Phytochemicals, such as polyphenols, minerals, enzymes, and sugars, can be extracted and are primarily bioactive compounds [[16], [17], [18]]. These compounds can be used in cosmetics, drugs, flavors, and perfumes [19,20]. By implementing waste management, the cocoa processing industry can not only reduce food waste but also contribute to sustainability and create economic value [21].

CPH has the potential to be exploited with a primary emphasis on food applications, along with additional considerations for non-food applications. CPH dietary fiber has been applied to several food products such as emulsion type pork sausages [22], high-fiber bread [23], and muffins [24]. The study of Adi-Dako et al. (2016) [25] showed that CPH pectin has dose-dependent moderate antibacterial activity against *S. aureus, P. aeruginosa, B. subtilis, E. coli, Salmonella typhi,* and *Shigella* spp. CPH pectin may function similarly to citrus pectin, which has been shown to be an effective probiotic encapsulating agent [26]. Santana et al. (2018) [27] utilized CPH to make xylitol with a yield of 0.52 g/g and a fermentation efficiency of 56.6 %, which effectively stimulated the metabolism of the immune, digestive, lipid, and bone systems [28]. CPH also showed potential as an inert substrate for the biotransformation of secondary metabolites by fungal strains, namely volatile fragrance elements [29]. The gel derived from the CPH extract lowered in vivo skin wrinkles, and after three weeks of treatment, skin moisture also increased [30]. Commercial cocoa pod ash filtrate is a component of African black soap, which is then utilized in conditioning washing cream that is devoid of harmful chemicals [31].

CPH is abundant in fiber (particularly lignin, cellulose, hemicellulose, and pectin), as well as antioxidants (phenolic acids, etc.) [32,33]. CPH has the potential to be used as a source of pectin [34,35]. The most common method of extracting pectin from CPH is acid extraction using organic acids such as citric acid, acetic acid, and oxalic acid. In recent years, green extraction methods have been developed and used to extract pectin [36]. Although citric acid, acetic acid, and oxalic acid are considered relatively environmentally friendly, the term "green extraction" usually refers to techniques that reduce environmental impact and increase sustainability, that promising less energy, take shorter time, produce fewer by-products, higher yields, and using renewable techniques [[37], [38], [39]]. Green extraction methods cover a range of techniques that expand beyond the selection of solvents. The green extraction methods are considered as more environmentally friendly compared to the utilization of conventional organic acids due to their incorporation of innovative technologies that enhance the efficiency and sustainability of the extraction process.

The main objective of this review is to provide a systematic study of the potential uses of cocoa by-products, notably focusing on CPH as the most abundant component, as well as emerging green technology approaches to extract pectin and overcome upcoming challenges such as waste management. These insights provide essential groundwork for food manufacturers and researchers to uncover novel avenues for cocoa by-products, especially CPH, within the framework of a sustainable society.

### 1.1 Cocoa pod husk

**Fig. 1:**
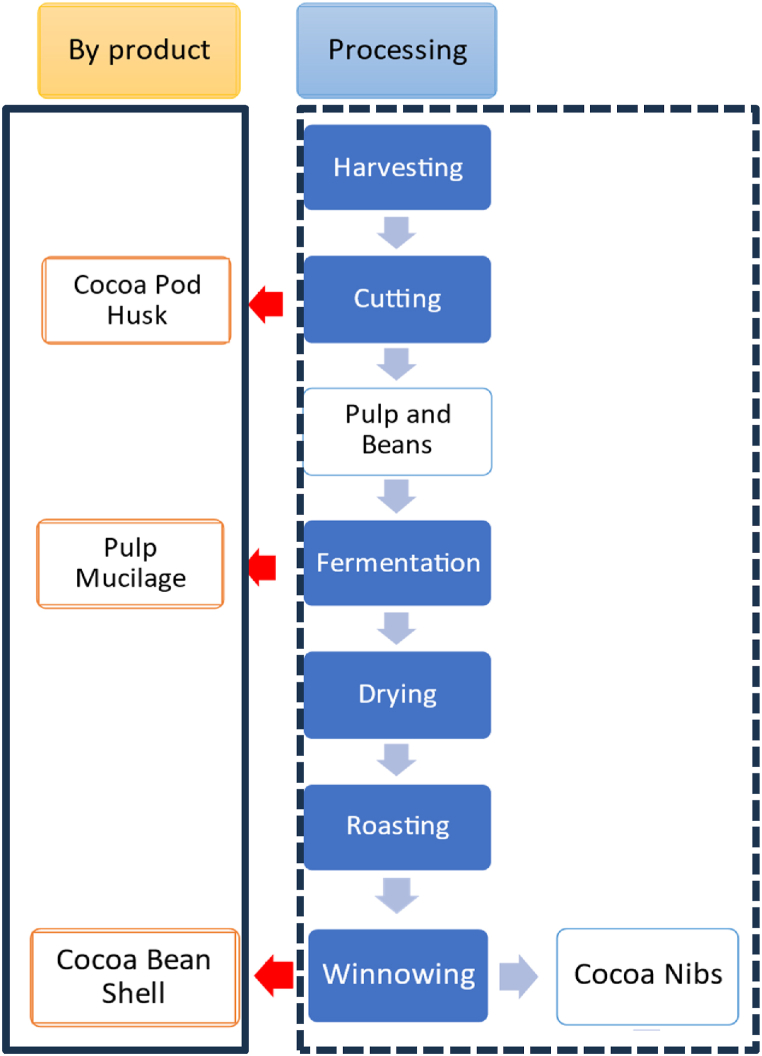
Flowchart of primary cacao processing, including commercial products and by-products. Adapted from Vandenberghe et al. [41].

**Fig. 2:**
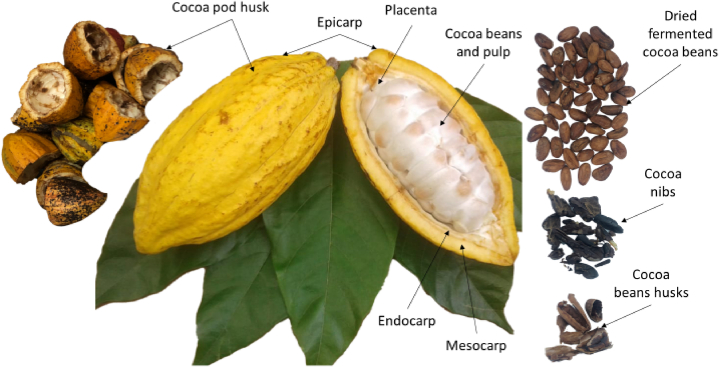
Parts of the cocoa fruit. Adapted from Ouattara et al. [42].

Fig. 1 show the primary cacao processing, including commercial products and by-products. Upon reaching maturity, the pods are picked and opened with a club, knife, or machete, which necessitates substantial skill and expertise to prevent seed damage [11]. The initial process is the spontaneous fermentation of cocoa beans which is an important phase for removing the pulp while producing flavor precursors for chocolate [40]. Subsequently, the drying, roasting, and crushing processes are implemented until cocoa nibs are obtained, which are subsequently utilized in the production of chocolate. The entirety of the parts of the cocoa pod can be seen in Fig. 2.

CPH is often disposed of as an organic fertilizer in cocoa farms, a procedure that delivers organic matter to the soil and permits the release of nutrients and their conversion to plant-available forms following decomposition [43]. This practice can have an adverse effect on the environment and is the main contributor to fungal diseases that damage cocoa plants, like black fruit rot [44]. Environmental harm and financial losses will follow if these waste items are not removed. Waste from cocoa pods has an adverse effect on the environment because bacteria produce methane and carbon dioxide while degrading the waste [45].

### 1.2 Physicochemical properties of CPH

The CPH consists of various layers, including the endocarp, epicarp, mesocarp and sclerotic component. It possesses a rounded, rough and generally thick look. The mesocarp has a hard composite structure that can keep cocoa beans in place even in harsh situations. The exocarp is the outside layer, while the endocarp is the inner section consisting of a soft, white tissue that retains cocoa beans inside a lubricated inner chamber [42]. Table 1 shows the previous study on the nutritional and fibre composition of CPH. Lu et al. (2018) [33] reported that fibrous compounds such as 19.7–26.1 % cellulose, 8.7–12.8 % hemicellulose, 14–28 % lignin and 6.0–12.6 % pectin are the primary constituents of the CPH. The endocarp is rich in pectic materials, the epicarp is richer in lignin, and the mesocarp consists primarily of cellulose [45]. Campos-Vega et al. (2018) [46] found that less than 1 cm of freshly cut CPH had 87 % organic matter, which consisted of fiber (55.7 %), nitrogen-free extract (20.6 %), crude protein (8.4 %), and fat (2.5 %). Additionally, the pretreatment applied to process CPH such as blanching or boiling has a direct impact on its chemical composition [32,46]. Yapo et al. (2013) [44] reported that the average weight of ripe cacao pods was 516.7 ± 1.9 g, with the average weight of raw cacao beans was 121.8 ± 1.7 g, while the produced husks made up 70.6 ± 2.4 % of the fresh pods and consisted predominantly of moisture (84.6 ± 2.4 %). The weight of the dried husks was assessed to be approximately 11.0 % of the weight of the fresh pods [47].

**Table 1:** Previous report on the fiber and nutrient of CPH.

Composition	Amount (%)	References
Moisture	79.7	Antwi et al. (2019) [48]
Ash	7.39	Antwi et al. (2019) [49]
Protein	5.9	Campos-Vega et al. (2018) [34]
Carbohydrates	49.3	Delgado-Ospina et al. (2021) [32]
Fat	0.79	Delgado-Ospina et al. (2021) [21]
Crude fiber	22.6	Campos-Vega et al. (2018) [34]
Cellulose	35.0	Campos-Vega et al. (2018) [34]
Hemicellulose	11.0	Campos-Vega et al. (2018) [34]
Lignin	14.6	Campos-Vega et al. (2018) [34]
Pectin	6.1	Campos-Vega et al. (2018) [34]
Mineral (% of total solid)		
N	1.42	Antwi et al. (2019) [49]
P	0.19	Lu et al. (2018) [22]
K	3.18	Campos-Vega et al. (2018) [34]
Na	3.1	Campos-Vega et al. (2018) [34]
Mg	0.22	Campos-Vega et al. (2018) [34]
Ca	0.30	Antwi et al. (2019) [49]
Mn (mg/kg)	50.10	Antwi et al. (2019) [49]
Zn (mg/kg)	56.23	Antwi et al. (2019) [49]

## 2 Cocoa pod husk as pectin source

Pectin is a complex polysaccharide consisting primarily of methoxy esterified d-galacturonic acid and rhamnose units. It also contains a significant quantity of neutral sugars, such as arabinose and galactose, as well as smaller amounts of other sugars [50]. Pectins are classified according to their methoxy composition and the rate at which they form gels. Pectins can roughly be categorised as either high methoxy pectins (>50 % esterified) or low methoxy pectins (50 % esterified) [51]. Low methoxy pectins generate partially shear-reversible gels [52]. Pectin is a significant element in food products because of its gelling, film-forming and thickening properties, which could give texture enhancement and stability in food products [[53], [54], [55]]. Pectins are organic polymers used as emulsifiers, gel-forming agents, thickeners, stabilizers, and fat or sugar substitutes [56]. Pectin is a naturally occurring component of all omnivorous diets and an important source of dietary fiber [57].

CPH pectin is classified as a low-methoxyl (LM) pectin due to its low methoxyl content, which ranges from 3 to 5% [58]. The degree of esterification (DE) of CPH pectin is found to be 26.8 ± 2.5 %, providing more evidence that it is LM pectin [59]. Muñoz-Almagro et al. (2013) [35] observed that galacturonic acid (GalA) was the predominant monosaccharide in CPH pectin, comprising 50–59 % of the total. Similarly, Chan and Choo (2013) [60] and Vriesmann et al. (2012) [61] achieved similar findings, with a range of 59–65 %, depending on the extraction technique employed. According to Priyangini et al. (2019), CPH pectin has high ash and protein content (~8 %), indicating that both extracts could be good vegetal sources of minerals and proteins for human nutrition. The CPH pectin showed a glass transition temperature (Tg) of 159.4 °C, a melting temperature (Tm) of 235.4 °C, and a melting enthalpy (ΔHm) of 68.35 J/g, indicating that the CPH pectin has good thermal stability and better than the pectin derived from citrus peel and apple pomace [58].

According to the Adi-Dako et al. (2016) [25] report, due to its favourable physicochemical characteristics, antibacterial activity, and high mineral, cocoa pod husk (CPH) pectin has potential usage as a flexible pharmaceutical excipient and nutraceutical. In aqueous conditions, the pectin displayed pseudoplastic, shear thinning behaviour, and a high capacity for swelling, making it an excellent candidate for use as a medicinal excipient. The pectin also shown modest antibacterial activity against both gram-positive and gram-negative microbes, suggesting that it may have applications as a natural antimicrobial agent. CPH pectin exhibited the highest MIC against *E. coli* among gram-positive bacteria. CPH pectin has less efficacy against gram-positive bacteria than gram-negative bacteria. Additionally, it was discovered that CPH pectin is a rich source of minerals such as sodium, magnesium, calcium, iron, potassium, phosphorus, and sulphur, making it a potential nutraceutical.

## 3 Waste management of cocoa pod husk by product

The majority of cocoa by-products are generated during the primary processing stage, which is carried out mainly by cocoa farmers. Typically, chocolate manufacturers purchase only dry fermented cocoa beans from farmers. A large number of cocoa byproducts, including CPH, pulp, and mucilage, need to be handled by cocoa farmers. The inadequate availability of resources, skills, and technology among cocoa farmers affects their ability to effectively manage waste generated from cocoa production. Consequently, cocoa by-products are typically handled in a simple way, including disposal in field, burial as compost, or use as animal feed. Based on Wee et al. (2017) [62] report, there are three main limitations to good governance in implementing Solid Waste Management: insufficient funding, inadequate staff competency, and ambiguity in the policy implementation system. This is in line with the fact that the majority of cocoa producers are small farmers (95 %), who have limited funds and low competence in managing and utilizing cocoa by-products. Therefore, cocoa farmers can only use their agricultural by-products as fertilizer in landfills or as animal feed. An integrated and comprehensive system is required to collect by-products, manage, process and produce valuable compounds from cocoa by-products, in order to make operations more efficient and cost-effective.

CPH production shows significant temporal variability due to fluctuations in cocoa fruit processing rates, driven by annual crop yields. This causes seasonal variations in cocoa production, with the majority of cocoa harvest taking place from May to October and starting slowly in the minor crop season of November to April [63]. As a result, most CPH processing can only operate during this period. To manage this intra-annual variability, CPH must be stored before undergoing various valorization processes. Anoraga et al. (2022) [64] reported that CPH stored in the ambient room experienced serious damage with mold and brown color appearing quickly within a few days. Meanwhile, CPH was stored in the cool case (5 ± 1 °C) and freezer (−5±1 °C), mold growth appeared after seven days in the cool case and there was no mold in the freezer because of the presence of ice crystals. However, some browning occurs in the freezer.

The distribution of CPH from producers or farmers to CPH processors results in transportation costs which are influenced by factors such as road conditions, vehicle size and density of wet CPH. Increasing the dry matter content per unit volume through processes such as pressing or drying will reduce transportation costs, enable transportation over longer distances and expand utilization markets [65].

It has been previously mentioned., the possibility of valorization of CPH for the production of functional and valuable compounds for food ingredients has been evaluated through several studies [22,23]. However, currently, more research is needed to guarantee the food safety of food ingredients obtained from food waste or by-products. Fruit by products can contain potentially pathogenic nature, organic residues, high water content, as well as physical and chemical contaminants that may be a threat to health. Evaluation of safety and quality of food by-product utilization must be guaranteed as intended for human consumption. In Europe, the utilization of food by-products is subject to regulations based on their intended purpose. If the end product is intended for human consumption or serves as a food additive, the input material must consist of food or food ingredients that comply with the guidelines outlined in the European Commission (EC) Regulation No. 178/2002 [66]. In the case of nutraceuticals, the EU classifies these products as food supplements, and they are regulated as food under Directive 2002/46/EC. The basic principles of EU legislation on contaminants of food were laid down in the Council Regulation 315/93/EEC of February 8, 1993 [67].

Furthermore, the utilization of cocoa by-product aligns with the Sustainable Development Goal to achieve food security and improved nutrition (SDG2), especially empowering small farmers, ending rural poverty and ensuring healthy lifestyles [68]. This included to achieve the environmental management of all wastes throughout their life cycle and significantly reduce their release to air, water and soil to minimize their adverse impacts on human health and the environment [69]. SDG-2 pursues to "end hunger, achieve food security, improve nutrition and encourage sustainable agriculture.” SDG-2, which is directly tied to society, the economy and the environment, is critical to the overall success of the SDG agenda [70]. Utilizing cocoa by-products results in alternative products that can be used to enhance nutrition, quality and food security while also reduce environmental issues like landfill pollution.

One potential strategy for enhancing the sustainability of the cocoa food chain is the use of effective management practises for the residual biomass generated during the biotransformation process of cocoa pods. Implementing strategies to enhance this feature can effectively mitigate phytosanitary issues while concurrently generating additional revenue for farmers, by capitalising on the underutilized residual biomass. Utilizing biotechnology tools and extraction techniques can be a relevant and efficient method for obtaining value-added products from cocoa production chain by-products [11].

## 4 Green technique for cocoa pod husk utilization

The conventional extraction of pectin from fruit by products such as cocoa pod husk (CPH) is harmful to the environment and diminishes pectin quality [71]. After conventional acid extraction, pectin is degraded, reducing its purity and functionality. This conventional technique can eliminate volatile molecules that influence the flavor and aroma of pectin. In addition, acid extraction generates large quantities of waste, such as acid solutions and other residues, which can be harmful to the environment if not properly managed [39]. Using high temperatures and extended extraction times can consume energy and produce greenhouse gases [72].

Extraction is the first step to separate the desired natural products from the raw materials. The extraction of natural products progresses through the following stages: (1) the solvent penetrates into the solid matrix; (2) the solute dissolves in the solvents; (3) the solute is diffused out of the solid matrix; (4) the extracted solutes are collected. Any factor enhancing the diffusivity and solubility in the above steps will facilitate the extraction. The conventional extraction methods usually use chemical and require a large volume of solvents and long extraction time. Pectin extraction is a steady process involving the hydrolysis, isolation and dissolution of pectins from plant tissue [73].

Green extraction is based on the development of extraction methods that use less energy, permit the use of renewable natural resources and alternative solvents, and guarantee the production of safe and high-quality extracts and products [74]. There are three main alternatives to develop and implement green extraction on a laboratory and industrial scale, as well as to work toward an ideal consumption of raw materials, solvents and energy, such as process optimization and improvement, non-specialized equipment use and innovation in procedures and processes, as well as the identification of substitute solvents [75]. According to Chemat et al. (2012) [76], there are six principles of green extraction of natural products that industries and scientists should take into consideration when establishing an innovative and green label, charter and standard, as well as when reflecting on how to improve all attributes of solid-liquid extraction. These six principles are innovation through a variety of selections and utilization of renewable plant resource, utilization of alternate solvents, primarily water or agro-solvents, reduction of energy usage through energy recovery and the use of cutting-edge technologies, including the bio- and agro-refining industries in the production of co-products instead of wastes, minimize unit operations and prioritize secure, robust and governed processes, and seek a non-denatured, biodegradable and contaminant-free extract. The principles observed by scientists and through their successful implementation by the business sector have been acknowledged and explained not as rules, but as innovative examples to imitate.

Saving energy and money during the green extraction process is possible through careful energy management. It is possible to reduce the amount of energy needed for the extraction process by using ultrasonic, subcritical water, enzyme, and microwave aids, and by keeping temperatures as low as possible without sacrificing quality or yield. The use of these green technologies has been shown to increase extraction yields, decrease processing times, and lower energy needs when compared to conventional extraction methods [34]. Ethanol is used in excess to precipitate the pectin, wash it, and remove any impurities. Large amounts of solvent used in these techniques must be disposed of once they have delivered their technological purpose. Solvents can be recovered after a reaction by distilling or filtering the mixture [77]. The economic costs and environmental impact of extracting cocoa pectin necessitate the exploration of ways to recover, regain, reuse, or reduce ethanol.

A variety of single and combination extraction techniques for pectins have been developed. Enzyme, alkali and acid extractions are the most common types of single techniques. Combined techniques, such as subcritical extraction, microwave irradiation or ultrasound irradiation, can be used to increase extraction yield and reduce extraction time for pectins using conventional alkali, acid, and water procedures [78]. Table 2 shows the prevalent method for the extraction of CPH pectins, and also the comparison between conventional and green extraction methods. The extraction yield (mass of extract/mass of dry matter) is frequently used to show how the extraction conditions affect the extract [79]. The highest yield was obtained at 42 % through MAE as a green extraction method using power 300W for 30 min [80]. Muñoz-Almagro et al. (2019) [35] compared subcritical water extraction (SWE) to acid extraction. The results demonstrated that green extraction produced a higher yield (10.9 %). In addition, Hennessey-Ramos et al. (2021) [81] compared enzymatic, ultrasonic and acid extractions. Enzymatic extraction produced the highest yield. It was clear that the yield for the green extraction method was higher, coming in at 10.2 and 8.28 for enzymatic and ultrasound-assisted extraction, respectively.

**Table 2:** Extraction methods and parameters for cocoa pod husk (CPH) pectins.

Method	Extraction Condition		Solvent	Yield (%)	References
	Temperature (^o^C)/Power (W)	Time (min)			
**Conventional Method**					
Aqueous nitric acid	100 ^°^C	30	Aqueous nitric acid pH 1.5	9	[82]
Aqueous extraction	50 ^°^C and 100 ^°^C	90	Hot water	7.5 and 12.6	[53]
Aqueous citric Acid	95 ^°^C	95	Citric acid pH 3	10.1	[61]
Water, citric acid, and hydrochloric acid	95 ^°^C	30	Water, hydrochloric acid pH 2.5–4, and Citric acid pH 2.5 (maximum)	7.62	[60]
Water acidified	75 ^°^C	90	Nitric acid pH 2	8.6	[83]
Aquoeos Citric Acid	50 °C	–	Citric acid	23.3	[25]
Sugar acid	95 ^°^C	45	Ascorbic acid pH 2.5	4.2	[58]
Acid hydrolysis	85 ^°^C	90	Citric acid pH 3	6.18	[84]
Acid extraction	100 °C	150	Nitric acid pH 2.5	11.73	[85]
**Green Extraction Method**					
MAE	400 W	15	Oxalic acid pH 1.16	9.64	[86]
MAE	300 W	30	Citric acid pH 1.5, hydrochloric acid pH 1.6	42	[80]
**Comparison between Green Extraction and Conventional Method**					
SWE	121 °C 95 °C	30 95	Water Citric acid pH 3	10.9 8	[35]
Citric acid					
Enzymatic Ultrasound-assisted Citric acid	50 °C	18.54	Enzyme celluclast 1.5L	10.20	[81]
	60 °C	40	Citric acid pH 2.5	8.28	
	95 °C	95	Citric acid pH 2.5	8.08	

### 4.1 Microwave extraction

Microwave-assisted extraction (MAE) is a method that uses microwave power to generate solvents that are in contact with a sample in order to separate analytes from the sample matrix into the solvents. MAE makes it possible to quickly heat the sample solvent mixture, which is the main benefit of this method [87]. In MAE, microwaves are used to heat plant molecules in a process called dielectric heating. Non-ionizing radiation used in MAE causes ionic transport and dipole rotation. The combination of these two mechanisms produces intense heating. The heat helps speed up mass transfer and aids in the disintegration and release of pectins by causing the intricate pectin/cellulose/hemicellulose network to collapse [88,89]. Many researchers have recently explored MAE and discovered that it can significantly improve the yield and quality of extracted pectins [90,91].

Increasing microwave power increases pectin output by enhancing solvent penetration into plant matrix and delivering it to plant cells for pectin extraction. Molecular interaction with the electromagnetic field transfers energy quickly to the solvent and matrix, permitting component dissolution. Water may absorb microwave radiation and heat efficiently as a polar solvent. Microwave irradiation accelerates cell rupture through abrupt temperature rise and internal pressure increase inside plant cells, promoting sample surface degradation and pectin exudation into surrounding solvents [92].

Additionally, MAE can also lead to a decrease in the degree of esterification, resulting in an increase in the pectin's gel-forming properties. MAE has been shown to have a significant effect on the characteristics of fruit pectin. The increased extraction yield, alteration of molecular weight, and decreased degree of esterification are some of the key outcomes of MAE that can have important implications for the use of fruit pectin in various applications.

MAE affects the fruit pectin characteristic. MAE has also been shown to have an impact on the molecular weight and degree of esterification of pectin. The microwave energy can break down the pectin molecules and alter their molecular weight, which can result in the formation of low molecular weight pectins. Pectin extracted from fig skin using microwave-assisted extraction had a higher molecular weight than pectin extracted using citric acid extraction [93]. The degree of esterification of apple pectin extracted with microwave-assisted citric acid extraction was less than that obtained with conventional citric acid extraction. Acid hydrolysis of methyl ester groups may have been induced by the heat from microwave absorption and the low pH of the citric acid solution, leading to a low degree of esterification in the pectin [94]. Pectins extracted with MAE have homogalacturonan regions as their primary building block and galacturonic as their primary monosaccharide. Sweet lemon pectin extracted with the help of microwaves had a high galactose content [95].

### 4.2 Enzymatic extraction

The enzymatic extraction of physiologically active chemicals from plants is a viable alternative to solvent-based extraction techniques. Enzymes are suitable catalysts for extracting, altering or creating complex naturally occurring physiologically active substances [96]. Enzyme-assisted extraction is used to liberate chemicals that are bound on the inner surface and inaccessible via traditional solvent extraction [97]. Enzyme-assisted extraction is predicated on the intrinsic enzymes' capacity to activate reactions with high selectivity and the capacity to function under mild processing conditions in aqueous solutions [98]. The cell wall of a plant is made up of a complex structure of polysaccharides, including pectins. In the enzymatic extraction of pectin, cell wall degrading enzymes with minimal pectinolytic activity are utilized to hydrolyze non-pectin plant cell wall components [99]. Pectins can be extracted more efficiently and safely for the environment enzymatic method. In order to extract pectins, a variety of enzymes are utilized, including polygalacturonase, hemicellulose, protease and microbial mixed enzymes, cellulose, α-amylase, celluclast, alcalase and α-amylase and neutrase, Xylase, cellulose, b-glucosidase, endopolygalacturonase and pectinesterase [100].

Generally, enzymatic extraction was conducted for 4–24 h at 35–60 °C and pH 3.5–4.5 [[101], [102], [103]]. By hydrolyzing cellulose or hemicelluloses, enzymes break down the plant cell wall, releasing pectins that have been bound inside the intricate cellulose/hemicellulose network. The four enzymes, which are cellulase, protopectinase, hemicellulase and xylanase, are most frequently utilized in pectin extraction procedures. After 20 h, the yield of apple pomace pectin produced with cellulase was 7.2 %, which was much less than the yield produced with citric acid (23.3 %) [94]. Cellulase did not fully hydrolyze plant cell structure, limiting pectin production. Lam-inex C2K, a mix of cellulases, hemicellulases, xylanases and arabinoxylanases, releases pectin from plant cell walls. Enzyme-based extraction delivers high purity pectin and moderate processing temperatures, while decreasing equipment corrosion. Enzymatic lab-scale pectin extraction requires minimal concentrations. Enzymatic extraction is not normally appropriate for industrial scales with high raw material volumes and costs [95].

Pectin extraction is facilitated by enzymes, which break down the plant cell wall matrix and increase cell permeability. Enzyme-assisted extraction can result in a higher quality pectin, with improved gel-forming properties. This is due to the ability of enzymes to specifically target and break down the pectin molecules, resulting in a more homogeneous and purified product [104]. The addition of enzymes can also alter the molecular weight of the extracted pectin. Enzymes can break down the pectin molecules into smaller fragments, resulting in the formation of low molecular weight pectins [105]. The enzyme source and dosage, extraction time, and reaction pH and temperature all affect the molecular weight of pectin. The degree of esterification of the extracted pectin may change as a result of the addition of enzymes. The ester bonds in pectin can be broken by enzymes, which lowers the degree of esterification and improves the pectin's ability to form gels. Galacturonic acid was the most abundant monosaccharide in enzyme-extracted pectin [81,102,106]. In comparison to orange pectin obtained either through acid extraction, cellulase-extracted orange pectin had a greater proportion of rhamnogalacturonan I regions and more branched chains. In contrast to acid extraction, enzymatic extraction conditions are mild and do not compromise the pectin structure [106]. Pectins extracted with enzymes are highly methoxylated [94,103]. Enzyme-assisted extraction can have a significant impact on the characteristics of fruit pectin, including increased yield, improved quality, altered molecular weight, and changes in degree of esterification. These effects can have important implications for the use of fruit pectin in various applications.

### 4.3 Subcritical water extraction

Subcritical water extraction (SWE) is a new and strong technology at 100–374 °C and high pressure enough to preserve the liquid state [107]. SWE has become a common green extraction technology for the isolation of several kinds of substances from natural materials. The low price, safety and green character of water, high yields of target chemicals and reduced energy usage make this process suitable for prospective industrial applications [108]. SWE has an interesting and beneficial property that is as temperature rises, its polarity can dramatically decrease. This is because methanol or ethanol acts in the same way as SWE. SWE is now a green extraction agent for a variety of natural substances. From natural resource, SWE can simultaneously extract a number of active components. Liquid chromatography, gas chromatography, infrared spectrum and/or mass spectrometry are used to separate, identify and quantify each natural product substance in SWE [109]. The term "subcritical water" refers to liquid water that is maintained at temperatures above its typical boiling level without changing phases [110]. Temperature is the main determinant of pectin yields in SWE [111]. The hydrogen bond strength in the water diminishes when the temperature increases. The weakening of hydrogen bonds causes a decrease in water's dielectric constant and polarity. This decreases the amount of energy used to disrupt solute-matrix interactions and boosts pectin yields [112]. Moreover, when the temperature rises a particular threshold, yields may decline owing to pectin degradation [111]. Another important consideration in SWE is the extraction time because prolonged extraction might degrade pectins [113]. Pressure is also a critical part in SWE [114]. SWE has a number of benefits, including higher-quality extracts, faster extraction methods and the absence of acidic or alkaline solutions [115]. However, inadequate control of processing parameters can cause pectin hydrolysis, a major stumbling block for numerous commercial applications [111]. The use of SWE for industrial purposes has also been hampered by the equipment's relative complexity and expensive cost.

SWE can result in a higher quality pectin, with improved gel-forming properties. This is due to the ability of SWE to selectively extract the pectin without the presence of impurities and contaminants, resulting in a more pure and homogeneous product [116]. The increased temperature of the water offers several physical benefits, including increased diffusion, decreased viscosity, decreased surface tension, increased vapor pressures, and increased mass-transfer rates [113]. Due to an increase in hydrolysis reactions, the molecular weight of pectins extracted from apple pomace using subcritical water decreased significantly as the extraction temperature rose. The high temperature and pressure in SWE can break down the pectin molecules and alter their molecular weight, leading to the formation of low molecular weight pectins. Subcritical water extraction at high temperatures (100–140 C) produced citrus peel pectins with a lower molecular weight than conventional extraction methods. Extremely methoxylated apple and citrus pectins are obtained by subcritical water extraction [117]. SWE can also result in changes in the degree of esterification of the extracted pectin, leading to an increase in its gel-forming properties. Pectins extracted from pomelo using the same technique exhibited a degree of esterification between 29.7 and 45.5 %, indicating that they are lowly methoxylated [111]. In conclusion, SWE can have a significant effect on the characteristics of fruit pectin, including increased yield, improved quality, altered molecular weight, and changes in degree of esterification. These effects can have important implications for the use of fruit pectin in various applications.

### 4.4 Ultrasound-assisted extraction

Ultrasound-assisted extraction (UAE) is one of the most applicable techniques for large-scale industrial synthesis of natural compounds due to its rapid extraction rate, simplicity, higher yield, high efficiency, low cost and execution time. UAE can be used with other cutting-edge techniques, including MAE and enzymatic processing. Ultrasound generates constructive interference, which stimulates powerful physical pressures such as seismic waves and sonic streams to break plant elements cell membranes, minimize particle size and improve the interaction between target sugars and solvents [118]. A non-thermal method called UAE uses sonic energy to accelerate the release and diffusion of the target compounds by cavitating the solvent [119]. During UAE, the plant cell is broken up by cavitation, which makes it easier to get the pectin out. The power of the ultrasound is a key parameter that helps figure out how much pectins can be produced. Ultrasonic devices with low power have much lower yields than devices with high power. However, UAE has significant benefits over conventional heating techniques when used properly and with enough power [120]. These include accelerated extraction, better yields and improved extraction efficiency. Additionally, UAE tends to be safer, requires less energy and solvent [121]. Nevertheless, ultrasonic extraction can be inconsistent since the intensity of ultrasound waves decreases with distance from the emitter [122]. This can lead to inconsistencies in the standardization of different batches of pectins.

UAE has been found to increase the yield of pectin extraction compared to conventional methods. This is due to the ability of ultrasound to increase the solubility of pectin in the extraction solvent and release more pectin from the fruit tissue [123]. The high-frequency sound waves in UAE can break down the pectin molecules and alter their molecular weight, leading to the formation of low molecular weight pectins. Grapefruit pectin extracted using ultrasound had a lower molecular weight than pectin extracted using conventional methods. Ultrasound-assisted extraction of grapefruit pectins revealed that galactose, arabinose, and rhamnose made up the majority of the rhamnogalacturonan I region, while galacturonic acid was the primary component of the homogalacturonic acid region. In addition, the composition of rhamnogalacturonan I regions in grapefruit pectins was significantly greater than in pectins achieved through conventional extraction [124]. These findings indicate that, compared to conventional hot acid extraction, ultrasound-assisted extraction causes a more severe degradation of the homogalacturonan region. Pectins from grapefruit and apples that were extracted with ultrasound assistance were strongly eth-oxylated [94]. UAE can also result in changes in the degree of esterification of the extracted pectin, leading to an increase in its gel-forming properties. Ultrasound-assisted extraction yielded a low-methoxylated pectin from sour oranges due to the low degree of esterification present in the pectin [125]. The pectin chain was de-esterified by the high ultrasound power (150 W) and the extraction solution's low pH (1.5), resulting in a low degree of esterification value [35]. UAE can produce pectin of higher caliber and with better gel-forming abilities. This is because ultrasound has the ability to extract pectin selectively without the presence of impurities and contaminants, producing a more pure and uniform product.

### 4.5 Ultrasonic-microwave-assisted extraction

Ultrasonic-microwave simultaneous extraction (UMSE) is a revolutionary extraction method that is quick, effective and cost-effective. It combines ultrasonic and microwave technology to maximize the penetrating heating effect of microwave and the cavitation effect of ultrasonic [[49], [126], [127]]. A new technique for extracting pectins that lowers/prevents pectin degradation and improves extraction efficiency is ultrasonic-microwave assisted extraction [128]. While microwave radiation uniformly raises the system's temperature and speeds up cell-structure rupture, ultrasound cavitation destroys cell struvture and improves the mass transfer of pectins. The interaction of these effects facilitates the release of pectins from fruit cell membrane by dissolving complex networks of pectin, cellulose and hemicellulose [129]. As a result, UMSE produces pectins quickly and effectively at low temperatures, conserving energy and cutting costs. UMSE is currently in the experimental stages due to equipment limitations.

The pectin produced by UMSE has the potential to be of higher quality and exhibit enhanced gelling properties. Because UMSE can remove contaminants and impurities from the pectin extraction process, the final product is more pure and uniform. The high-frequency sound waves and high temperature in UMSE can break down pectin molecules and alter their molecular weight, resulting in the formation of pectins with a low molecular weight. Compared to jackfruit pectin, the molecular weight of pectin extracted from fig skin using ultrasonic-microwave technology was significantly higher [93]. This might be explained by the fig skin pectin's more intricate structure and higher percentage of galacturonic units. The most prevalent monosaccharide found after extraction of fig skin and pomelo peel pectins was galacturonic [128]. During the ultrasonic-microwave-assisted process, hemicellulose and amorphous cellulose were likely fragmented into glucose molecules [122]. The degree of esterification of extracted pectin can be altered by UMSE, resulting in an increase in its gel-forming properties. The ultrasonic-microwave-assisted extraction yielded a pectin from fig skin with a 32.4 % esterification degree, indicative of a poorly methoxylated product [93]. In conclusion, UMSE can have a significant impact on the properties of fruit pectin, including increased yield, enhanced quality, altered molecular weight, and alterations in the degree of esterification. These effects can have significant implications for the various applications of fruit pectin.

## 5 Conclusion

This review shows that CPH is an abundant, easily accessible, and renewable source of dietary fiber, nutraceuticals, functional foods, pectin, antioxidant compounds, theobromine, and minerals. Utilization of bioactive compounds from CPH can lead to the development of profitable basic products, potentially generating income for farmers and encouraging economic development. CPH contains several functional compounds that have the potential to be utilized in a variety of fields, including antibacterial, dietary fiber, xylitol sweetener, fragrance compounds, skin treatments, and pectin. Emerging green techniques for pectin extraction, such as enzymatic extraction and subcritical water extraction (SWE), promise higher yields, shorter time, and are environmentally friendly. This method has proven effective in extracting pectin from CPH, producing high-quality pectin with the desired functional properties. The development of efficient and environmentally friendly pectin extraction processes, coupled with appropriate waste management strategies, can help reduce the environmental impact of the cocoa industry and encourage a more sustainable approach to pectin production. It is hoped that the use of CPH for pectin extraction, along with the application of environmentally friendly technological approaches and effective waste management strategies, has great potential for the development of sustainable products and high-added value in the food and agricultural industry.

### Funding statement

This research was funded by UPM-SEARCA Scholarship Ref. GBG22-0923 and research grant No. GP-IPS/2023/9748700 (Grant Putra GP-IPS). We would like to thank Directorate of Research, Universitas Gadjah Mada, for the financial support for the Article Processing Charge (APC) payment.

### Data availability statement

This study is a review of scientific research papers, and all data comes from the research results in the reference literature.

## CRediT authorship contribution statement

**Satria Bhirawa Anoraga:** Writing – original draft, Visualization, Resources, Funding acquisition, Conceptualization. **Rosnah Shamsudin:** Writing – review & editing, Visualization, Funding acquisition, Conceptualization. **Muhammad Hazwan Hamzah:** Writing – review & editing, Methodology, Conceptualization. **Suzannah Sharif:** Resources, Conceptualization. **Arifin Dwi Saputro:** Writing – review & editing, Visualization, Resources, Funding acquisition, Conceptualization.

## Declaration of competing interest

The authors declare that they have no known competing financial interests or personal relationships that could be perceived as having influenced the work described in this paper.
